# Preservice physical education teachers’ professional action competence in education for sustainability: a mixed method research

**DOI:** 10.3389/fpsyg.2025.1601026

**Published:** 2025-06-25

**Authors:** Thomas Royet, Valérian Cece, Vanessa Lentillon-Kaestner, Jérémy Castéra, Olivier Vors

**Affiliations:** ^1^Haute École Pédagogique du Canton de Vaud, Lausanne, Switzerland; ^2^UMR7287 Institut des Sciences du Mouvement Etienne-Jules Marey (ISM), Marseille, Provence-Alpes-Côte d'Azur, France; ^3^UR 4671 ADEF – Apprentissage, Didactique, Evaluation, Formation, Faculté des Arts, Lettres, Langues, Sciences Humaines, Aix-Marseille Université, Marseille, Provence-Alpes-Côte d'Azur, France

**Keywords:** education for sustinability, preservice physical education teacher, professional action competence, latent profile analysis, mixed methods research

## Abstract

**Introduction:**

With the growing emphasis on sustainability, physical education (PE) teachers are expected to incorporate education for sustainability (EfS) into their teaching. Based on a mixed method, this study aimed to assess preservice PE teachers’ professional action competence in EfS (PACesd) and identify PACesd profiles.

**Methods:**

A total of 412 French preservice PE teachers completed a questionnaire measuring PACesd, along with open-ended questions enabling an external assessment of pedagogical content knowledge. Descriptive statistics, correlation analysis, and latent profile analysis were conducted to identify PAC profiles. Thematic analysis based on both qualitative and quantitative approaches was used on open-ended responses, allowing chi-square tests to identify differences across PAC profiles.

**Results:**

The results revealed moderate-high perceived pedagogical content knowledge and self-efficacy but low willingness to teach EfS. Four competence profiles emerged, with external assessment revealing key similarities and differences.

**Discussion:**

These results highlight the need for targeted professional development to support EfS integration in PE.

## Introduction

Integrating sustainability issues into education is an urgent necessity to address the pressing social, economic, and environmental challenges of the 21st century (UNESCO, 2022). Sustainability is a multidimensional concept that encompasses ecological, social, economic, political, and health-related dimensions, all intricately interlinked (Lohmann and Goller, 2023). A widely accepted definition from the Bruntland Report frames sustainability as “*meeting the needs and aspirations of the present generation without compromising the ability of future generations to meet their needs.*” (Bruntland, 1987, p. 292). This report—officially titled *Our Common Future*—was commissioned by the United Nations in the 1980s in response to growing global concern over environmental degradation and persistent inequalities. In a context aimed at proposing a comprehensive environmental strategy, this definition of sustainability emphasized the principle of intergenerational justice and the necessity of balancing socioeconomic development with environmental preservation. Based on Lohmann and Goller (2023) analysis, sustainability must also be understood across spatial scales, from local communities to global governance, and across temporal scales, considering both immediate actions and long-term consequences. Addressing sustainability challenges requires a dual approach that combines individual behavioral changes with structural transformations in policies, institutions, and economic systems (Fischer et al., 2012). By integrating these multiple dimensions and levels of action, societies create resilient and equitable pathways toward a sustainable future.

It is imperative to increase awareness and educate generations to facilitate the construction of a more sustainable world. While the need to include education for sustainability (EfS) in all school subjects is rarely questioned in contemporary research, the theoretical framework underlying EfS is fragmented. An emancipatory definition of EfS is often opposed to an instrumental definition (Wals et al., 2008). In an emancipatory approach, EfS is characterized by a focus on capacity building and critical thinking rather than an emphasis on instrumental goals such as directly changing learners’ behaviors (Wals, 2011). In contrast, an instrumental approach of EfS posits that the role of education is to modify ways of thinking and behaving that are viewed as unsustainable by instructors, curriculum designers, administrators, and society at large (Ribó, 2023). Historically, a focus on a content-based approach has been replaced by a focus on learning outcomes (Wiek et al., 2011), thus rendering emancipatory EfS the predominant institutional and scientific approach in the contemporary world. Indeed, an emancipatory definition of EfS is in line with the definition provided by UNESCO, which highlights the need to develop students’ knowledge, skills, and values and to empower them to meet both current and future global challenges, which are interconnected (Rieckmann, 2017). Specifically, the need for an emancipatory approach has also been advocated (Arias-Maldonado, 2022; Torsdottir et al., 2024; Wals et al., 2008). Even if further studies on this topic are needed, the effectiveness of an emancipatory approach has been demonstrated with respect to students’ sustainability consciousness (de Boeve- Pauw et al., 2015). In an emancipatory approach, the objective of EfS is to cultivate skilled and active citizens who are informed and motivated to live sustainably and to contribute to the development of a more sustainable society (Carbach and Fischer, 2017). In other words, the learning objectives associated with this approach can be summarized as focusing on the development of students’ action competence in terms of sustainability (Olsson et al., 2020; Sass et al., 2020).

**Figure 1:**
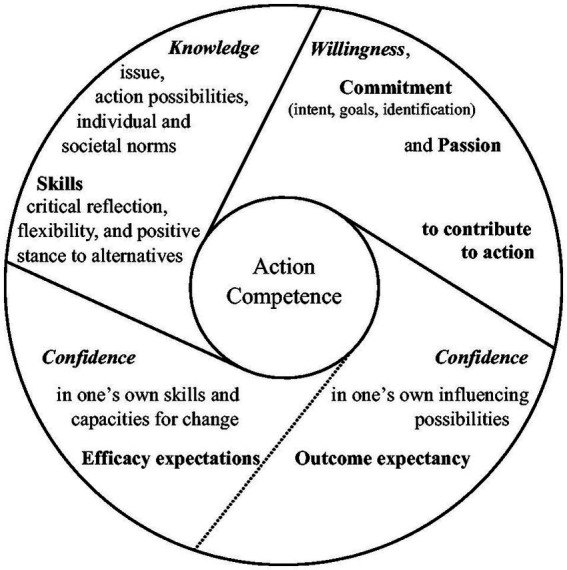
Core features of action competence based on Sass et al. (2020).

Action competence refers to self-determined actions that seek to help resolve problems related to sustainability (Sass et al., 2020). The action competence model is divided into three elements: knowledge related to the issue at hand, willingness to engage in action, and confidence in one’s ability to influence the identified opportunities (Mogensen and Schnack, 2018) (Figure 1). In this approach, action is defined as voluntary and goal-directed behavior that is intended to promote change or solve a specific problem (Breiting and Mogensen, 1999). The development of students’ action competence in terms of sustainability is therefore considered, in this context, a learning objective within the frame of sustainability education sequences. While all school subjects are designed to be integrated into a cross-curricular approach, the focus on action is particularly interesting in the context of physical education (PE), in which motor action is central. The singular way in which PE could contribute to the emergence of an emancipatory form of EfS, particularly with respect to the development of students’ action competence in terms of sustainability, remains unclear and poorly studied (Baena-Morales and Gonzalez-Villora, 2023; Fröberg et al., 2023; Royet et al., 2024). Nevertheless, explicit connections are established between a holistic approach to PE, including physical, cognitive, affective and social education, and the three dimensions of sustainability (i.e., ecological, social, and economic) (Baena-Morales and Gonzalez-Villora, 2023). Additionally, previous theoretical research has focused on fostering the development of critical and systems thinking in PE with the objective of promoting EfS (Baena-Morales et al., 2023b). Finally, recent studies using behavioral science approaches have highlighted the role of emotion in promoting sustainable actions (Brosch and Steg, 2021). By engaging the affective and sensory body, PE could provide a sensory and artistic pathway for teaching EfS (Heinrichs, 2021; Paintendre et al., 2021). PE teachers’ voices have also been heard with the aim of identifying how PE might fit with sustainability-related content (Fröberg et al., 2023). While some teachers have emphasized the importance of integrating EfS into PE (Lohmann et al., 2023), others are more skeptical of the idea of integrating sustainability-related content into their teaching (Lorente-Echeverría et al., 2024). In particular, teachers identified a lack of competence in the task of incorporating EfS into PE (Baena-Morales et al., 2022; Froberg et al., 2022; Lohmann and Goller, 2023), which could hinder the effective implementation of EfS. Therefore, PE teachers must acquire and integrate EfS-specific aspects of professional competence (Lohmann et al., 2021).

The importance of teachers’ professional competencies in EfS has been established, particularly with respect to learning outcomes for students (de Boeve- Pauw et al., 2015; Stevenson et al., 2017). However, several theoretical frameworks have been proposed as means of organizing and summarizing these professional competencies (e.g., “key sustainability competencies” or “curriculum, sustainable development, competencies, teacher training”) (Lohmann et al., 2021). Among these frameworks, in line with the action competence concept, professional action competence in the implementation for education for sustainable development (PACesd) focuses on teachers’ confidence in their capacities, willingness, (pedagogical content) knowledge, and skills pertaining to the implementation of EfS (Sass et al., 2022). In summary, to implement an emancipatory form of EfS effectively according to the PACesd framework, teachers should be familiar with pedagogical content knowledge (PCK) related to sustainability and EfS. They should also be willing to implement EfS in their teaching and exhibit high levels of perceived self-efficacy with respect to their ability to implement EfS. The development of teachers’ PAC could enable them to establish a powerful learning environment that can encourage the development of students’ action competence in terms of sustainability (Sinakou et al., 2019). An effective pedagogical environment in the context of an emancipatory form of EfS should offer students a holistic understanding of sustainability issues and the opportunity to express different opinions and solutions; furthermore, it should empower them to play an active role in the solutions thus considered (Sinakou et al., 2019; Torsdottir et al., 2024).

Nevertheless, the literature on PE teachers’ professional competences in the sustainability or EfS context highlights that while teachers exhibit a high level of self-efficacy regarding the task of integrating sustainability-related content into PE, they also exhibit a low level of sustainability PCK and an unclear level of willingness to implement sustainability in the context of PE. With respect to PCK in the context of EfS, PE teachers exhibit certain misunderstandings and misconceptions (Baena-Morales et al., 2022; Lohmann and Goller, 2023; Merma-Molina et al., 2023). PE teachers’ perceptions of sustainability are characterized by a certain degree of vagueness and inaccuracy (Merma-Molina et al., 2023). Nevertheless, in terms of self-efficacy in the process of implementing sustainability-related content in the context of PE, teachers seem to exhibit high self-perception levels of competence (Baena-Morales et al., 2023a; Froberg et al., 2022; Wiklander et al., 2024). Finally, PE teachers’ willingness to implement EfS in the context of PE seems to be unclear. Although some studies have highlighted that PE teachers find it important to implement EfS and work toward the development of sustainable institutions (Lohmann and Goller, 2023; Lohmann et al., 2023), Lorente-Echeverría et al. (2024) highlighted the fact that a high percentage of future PE teachers exhibit negative perceptions of sustainable development. Thus, while the professional competences that have been identified within the PACesd framework have been studied separately, no study has used the same sample to investigate the willingness, PCK and self-efficacy of PE teachers in the specific context of an emancipatory form of EfS in the context of PE classes.

Several tools have been developed to evaluate PE teachers’ professional competencies in the context of EfS or sustainability. The Physical Education for Sustainable Development instrument (Baena-Morales et al., 2024) was designed to measure the capacity of PE teachers to improve their skills and attitudes with the aim of supporting sustainable development. Lohmann et al. (2023) developed a scale that captures PE teachers’ beliefs regarding the relevance of sustainable development, both generally and in the particular context of PE. While the first tool is not part of the specific context of ESD, the second measures only teachers’ beliefs about implementing ESD in PE. These two PE contextualized instruments are therefore insufficient for evaluating the ESD action competences identified above. In addition, in the PE context, several scales have been validated to facilitate the measurement of a particular professional competence, such as teachers’ confidence in their capacities (Effeney and Davis, 2013; Malandrakis et al., 2019) or knowledge (Koch et al., 2013). Nevertheless, to our knowledge, the PACesd questionnaire (Sass et al., 2022) seems to be the only instrument used to measure PACesd as a whole. It was designed to measure teachers’ professional action competence in the process of implementing EfS in primary and secondary schools across different school subjects. In line with the concept of action competence, this questionnaire appears adapted to enable an effective investigation of professional competence to teach EfS in PE. However, the literature on teaching competencies has already demonstrated the difficulties teachers face in self-assessing their PCK, leading to significant differences between perceived PCK and an external assessment of PCK (Maderick et al., 2016). In addition, self-reported data on PCK in EfS, collected through the PACesd questionnaire, offer valuable insights, although they may be subject to biases such as social desirability or inaccuracies in self-perception (Tempelaar et al., 2020). To ensure a more reliable and comprehensive assessment, it is essential to complement these subjective measures with an external evaluation of preservice PE teachers’ actual competence in these areas (Sass et al., 2022). An external assessment can provide objective verification, identify potential gaps between perceived and actual professional competence, and offer a more nuanced understanding of preservice PE teachers’ PCK in EfS competence. This triangulated approach enhances the validity of findings and supports more informed decision-making in curriculum development and teacher training.

This study has two main objectives: (1) to evaluate preservice PE teachers’ professional action competence (PAC) in the process of implementing EfS and (2) to identify profiles to which preservice PE teachers belong based on their levels of PAC in the process of implementing EfS.

This study is relevant for three main reasons. First, the literature on PE teachers’ professional competencies in the process of implementing EfS (PACesd) remains scarce. No study has measured PACesd as a whole in the specific context of PE. To support the development of an emancipatory form of EfS in the context of PE, an initial assessment of preservice PE teachers’ PAC seems to be essential. In this case, mixed method research seems relevant for conducting an accurate assessment of teaching competences, particularly regarding PCK. To our knowledge, such an approach has never been used in the specific context of assessing the competences of preservice PE teachers to teach EfS.

Second, investigating preservice teachers can allow us to obtain interesting knowledge regarding the acquisition of professional skills, especially with respect to how EfS pedagogical beliefs and practices develop during the initial stages of a teaching career (Girardet, 2018). Our focus on preservice teachers also allows us to assess the effectiveness of initial teacher training programs, thereby identifying potential gaps between social or institutional expectations and classroom realities (Borko and Putnam, 1996). Additionally, studying this population offers a forwards-looking perspective that can anticipate future needs and trends in education as new generations of teachers navigate evolving societal demands (Borko and Putnam, 1996). This task is particularly relevant with respect to EfS, which emerged relatively recently as an expectation within educational frameworks and remains in constant evolution as scholarly and global priorities and challenges continue to shift.

Third, an exploration of profiles through person-centered approaches in the context of a study on preservice PE teachers’ competencies seems to be necessary, as this population is far from homogeneous although members of this group share a common stage in terms of their career trajectory (e.g., Fives et al., 2014). Future teachers incorporate various backgrounds, beliefs, and motivations into their training, which could influence the ways in which they engage with and develop teaching skills (Akkerman and Meijer, 2011). They also have a personal sensitivity to sustainability issues (Van der Werff et al., 2013). Previous studies have highlighted the high level of heterogeneity that characterizes teachers’ interest in EfS (e.g., Sinakou et al., 2024). This approach enables targeted interventions to be incorporated into teacher education programs, thereby ensuring that support and training are designed to suit the specific needs and strengths of different subgroups; this process ultimately leads to more effective professional development and better implementation.

## Materials and methods

### Participants

A total of 412 French preservice PE secondary school teachers (*M*_age_ = 22.61 years, SD = 2.24; age range: 20–44 years) participated in this study. Participants were recruited on a voluntary basis from eighteen teacher training institutions. One hundred forty-one participants were female, whereas 271 were male. The participants were required to be in the final 2 years of their training before becoming in-service teachers and to be engaged in teaching internships.

### Measures and procedure

The participants were informed that they could withdraw from the study at any time. They provided written informed consent in line with the requirements of the Declaration of Helsinki (Association WM, 2001). The participation of the preservice teachers was entirely voluntary. We informed them that their responses would be confidential. Preservice teachers were contacted by their local instructor with the approval of the relevant institution. Recruitment occurred during April 2024.

A cross-sectional and mixed method approach was used to assess the preservice PE teachers’ PACesd. First, the participants were asked to answer the PACesd questionnaire (Sass et al., 2022) (Appendix 1). They completed the questionnaires individually via an online format; the questionnaire completion process took approximately 15 min. The formally translated version of the PACesd questionnaire consists of 32 items used to measure action competence in terms of three factors: perceived pedagogical content knowledge regarding EfS (pPCKesd) (11 items, e.g., “*I am confident that as a teacher I can formulate learning objectives for my students regarding sustainable development*”); self-efficacy regarding EfS (SEesd) (11 items, e.g., “*I am confident that as a teacher I can develop students’ ability to understand the interconnectivity between the social, environmental and economic aspects of sustainable development*”); and willingness for EfS (Wesd) (10 items, e.g., “*I try to plan my daily work so that I have as much time as possible to spend on EfS*”). The questionnaire was translated via a back-translation method that involved two native speakers (Beaton et al., 2000). Initially, the questionnaire was translated from its original language into the target language by one bilingual translator. Another bilingual translator, who was blind to the original version of the questionnaire, subsequently translated the questionnaire back into the original language. This process ensures that any discrepancies or ambiguities that emerged during the translation process could be identified and corrected, thereby enhancing the accuracy and equivalence of the translated version of the questionnaire. The participants responded to the items included in the questionnaire on a six-point Likert scale ranging from 1 (not at all) to 6 (completely).

**Figure 2:**
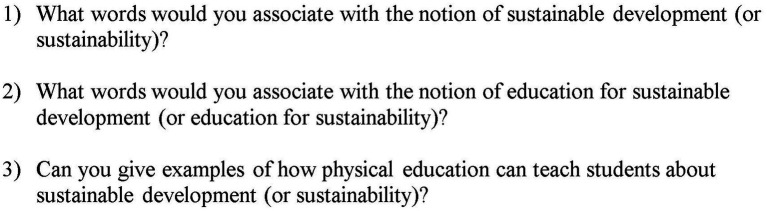
Open-ended questions asked to pre-service physical education teachers.

Second, the participants were also required to respond to three open-ended questions (Figure 2), which were designed to assess their understanding of the sustainability and EfS concepts and their application in PE. The answers to these questions made it possible to assess both the teachers’ understanding of content related to the concepts of sustainability and EfS, as well as their teaching methods. In this sense, these open-ended questions served as an external assessment of the preservice teachers’ PCK, complementing the self-reported PACesd questionnaire.

### Data analyses

The initial analyses were performed via the R program (version 4.4.1). We began this process by conducting a psychometric evaluation of the questionnaire, the French version of which has never been validated for use among PE teachers. Model fit was assessed in terms of Cronbach’s *α* coefficient, the comparative fit index (CFI), the Tucker–Lewis index (TLI), the root mean square error of approximation (RMSEA) and the corresponding confidence interval (90% CI), the standardized root mean square residual (SRMR), and the chi-square test of model fit (Hu and Bentler, 1999). A good fit was indicated by a CFI and TLI greater than 0.90 (>0.80 was sometimes viewed as acceptable) and a RMSEA and SRMR less than 0.08 (Hu and Bentler, 1999).

We subsequently conducted preliminary analyses, which included descriptive statistics (means, standard deviations, etc.) and correlation analyses among the various factors associated with the PACesd questionnaire. A one-sample t test was conducted to explore potentially significant differences in the mean scores of the factors in comparison with the sample used by Sass et al. (2022) in their questionnaire validation study.

The subsequent analyses were conducted via Mplus Version 7.3 software (Los Angeles, CA, USA). For all the models, the full information maximum likelihood (FIML) estimation method was employed to address missing data. FIML has been identified as a more efficient and unbiased approach (under the missing-at-random assumption) than listwise deletion, which can produce biased parameters (Enders, 2022). In this study, we used a latent profile analysis (LPA) approach. Latent analyses assume that a latent class variable can be inferred from a combination of various indicators (Lanza et al., 2010). Although no strict rules have been proposed regarding the required sample size in latent analyses, Collins and Wugalter (1992) suggested a minimum sample size of “somewhat smaller than 300.” This method facilitates the identification of subgroups that exhibit similar scores and relationships among the dimensions of PACesd. First, we selected a model that accurately captured the number and characteristics of the profiles based on the Wesd, pPCKesd, and SEesd scores. We computed a series of models that featured an increasing number of profiles (from 1 to 5) to determine which model exhibited the best fit (Cece et al., 2021; Lanza et al., 2010). A combination of statistical indicators was used to identify the best-fitting model. These indicators included the log likelihood value, the Akaike information criterion (AIC), the Bayesian information criterion (BIC), the adjusted BIC (ABIC), entropy, and the Lo, Mendell, and Rubin (LRT) likelihood ratio test. The model that exhibited the smallest AIC, BIC, and ABIC values, alongside the highest log likelihood and entropy values, was considered to exhibit the best fit. In latent analyses, reliance on a single indicator is insufficient; rather, when interpreting the LPA results, it is crucial to consider a combination of statistical indicators as well as the substantive meaning of each emerging profile (Lanza et al., 2010). The profiles were analyzed based on their scores on each dimension of Wesd, pPCKesd, and SEesd.

**Figure 3:**
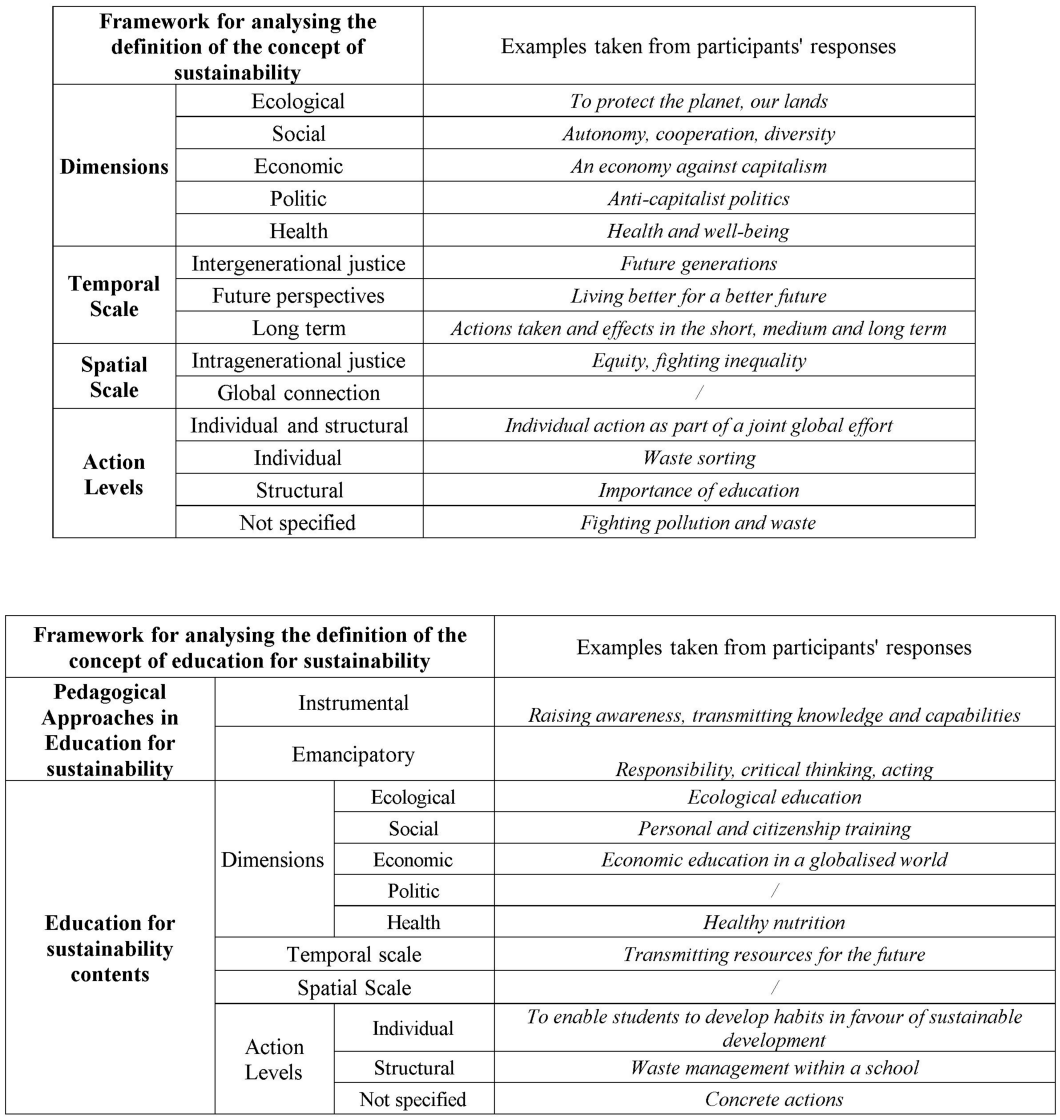
Definition frameworks (inspired by Lohmann and Goller, 2023) used for the deductive thematic analysis of the answers to the open-ended questions concerning the concepts of sustainability and education for sustainability and examples taken from participants’ responses.

The three open-ended responses were analyzed manually and thematically via both inductive and deductive methods (Fereday and Muir-Cochrane, 2006) and quantified (Guest et al., 2012). A subset was processed independently by the first two authors. A pooling process was used to verify the consistency of the deductive (questions 1 and 2) and inductive (question 3) thematic analyses. Points of disagreement were discussed in order to find points of convergence. This process improved the replicability of the analyses performed. For the first two questions, predefined analytical frameworks inspired by Lohmann and Goller (2023) were employed for deductive analysis (Figure 3). The first framework defines the concept of sustainability, as presented above, around different dimensions (ecological, social, economic, health, political), spatial and temporal scales, and different levels of action for acting. The second framework defines the concept of EfS, focusing on the content of sustainability and emphasizing the educational model (emancipatory or instrumental). The aim of the deductive analysis was to identify, in the participants’ answers, the concordances with the definitional frameworks used. The greater the degree of concordance between the participants’ answers and the framework was, the greater the importance of the level of PCK. For the third question, a combined approach was used: deductive analysis based on the EfS definition framework and inductive thematic analysis to extract emerging dominant themes (Fereday and Muir-Cochrane, 2006). The aim of this question was both to identify an understanding of the concept of EfS in the context of PE (deductive analysis) and to identify the content emphasized by future teachers in this specific field (inductive analysis). For all three questions, the results of the thematic analysis were quantified, enabling an exact count of occurrences for each framework element (deductive analysis) or theme (inductive analysis) across all participants (Guest et al., 2012). Initially, a global analysis of all the responses was conducted for the three questions. Subsequently, the quantification of responses allowed for independent sample chi-square tests (McHugh, 2013) to explore the significance of differences in responses between profiles. First, chi-square tests were performed to explore differences in responses across all profiles. We assumed that the distribution of expected cell frequencies was generally adequate for chi-square analysis. In cases where expected counts were below conventional criteria (e.g., <5), results were interpreted with caution. Second, if the chi-square value indicated a significance level < 0.10, in-depth comparisons between each profile were conducted using Bonferroni correction.

## Results

### Preliminary analyses

The Cronbach’s *α* coefficients consistently indicated internal consistency across the scales for SEesd (α = 0.858), Wesd (α = 0.900), and pPCKesd (α = 0.930). Additionally, the model fit indices exhibited satisfactory results; namely, the RMSEA (0.078) and SRMR (0.064) both fell within acceptable ranges, thus confirming that the model exhibited a good fit to the data. Although the CFI and TLI were below the ideal threshold (CFI = 0.837, TLI = 0.824), they were within an acceptable range for the use of the initial translated scale. Given the overall acceptability of the psychometric properties and the importance of capturing the constructs measured in this context, we proceeded to use this questionnaire in the study.

The means, standard errors, skewness, kurtosis, and correlation coefficients of the scales are presented in Table 1. All variables showed skewness and kurtosis values within ±1, suggesting an approximately normal distribution suitable for parametric analyses. Moreover, intercorrelations among the Wesd, pPCKest, and SEesd were all below 0.70, which support the absence of problematic multicollinearity.

**Table 1:** Descriptive statistics and correlation coefficients.

Dependant variables	Descriptive statistics				Correlation coefficents			
	Mean	SD	Skweeness	Kurtosis	SEesd	pPCKesd	Wesd	IMPesd
SEesd	4.36	0.71	–0.29	0.71	–			
pPCKesd	4.35	0.78	–0.25	0.09	0.70***	–		
Wesd	3.09	0.92	0.03	–0.05	0.44***	0.52***	–	
IMPesd	4.69	0.89	–0.27	–0.24	0.42***	0.54***	0.46***	–

SEesd = Self-efficacy regarding education for sustainable development; pPCKesd = Perceived Pedagogical Content Knowledge; Wesd = Willingness for ESD; IMPesd = Importance for ESD.

A one-sample *t* test was conducted to compare the mean scores on Wesd, pPCKesd, and SEesd with the reference scores reported by Sass et al. (2022). The results indicated that the mean score for pPCKesd was significantly higher than the scores reported by Sass et al. (2022), *t*(411) = 2.27, *p* = 0.023, whereas the corresponding score for Wesd was significantly lower, *t*(411) = −4.98, *p* < 0.001. The scores for SEesd did not significantly differ from those reported by Sass et al. (2022), *t*(411) = −0.463, *p* = 0.644.

The correlation analyses revealed positive and significant associations among all the different dimensions of PAC. Specifically, SEesd was associated with Wesd (*r* = 0.44, *p* < 0.001), pPCKesd (*r* = 0.70, *p* < 0.001), and IMPessd (*r* = 0.42, *p* < 0.001). Similarly, pPCKesd was positively associated with Wesd (*r* = 0.52, *p* < 0.001).

**Figure 4:**
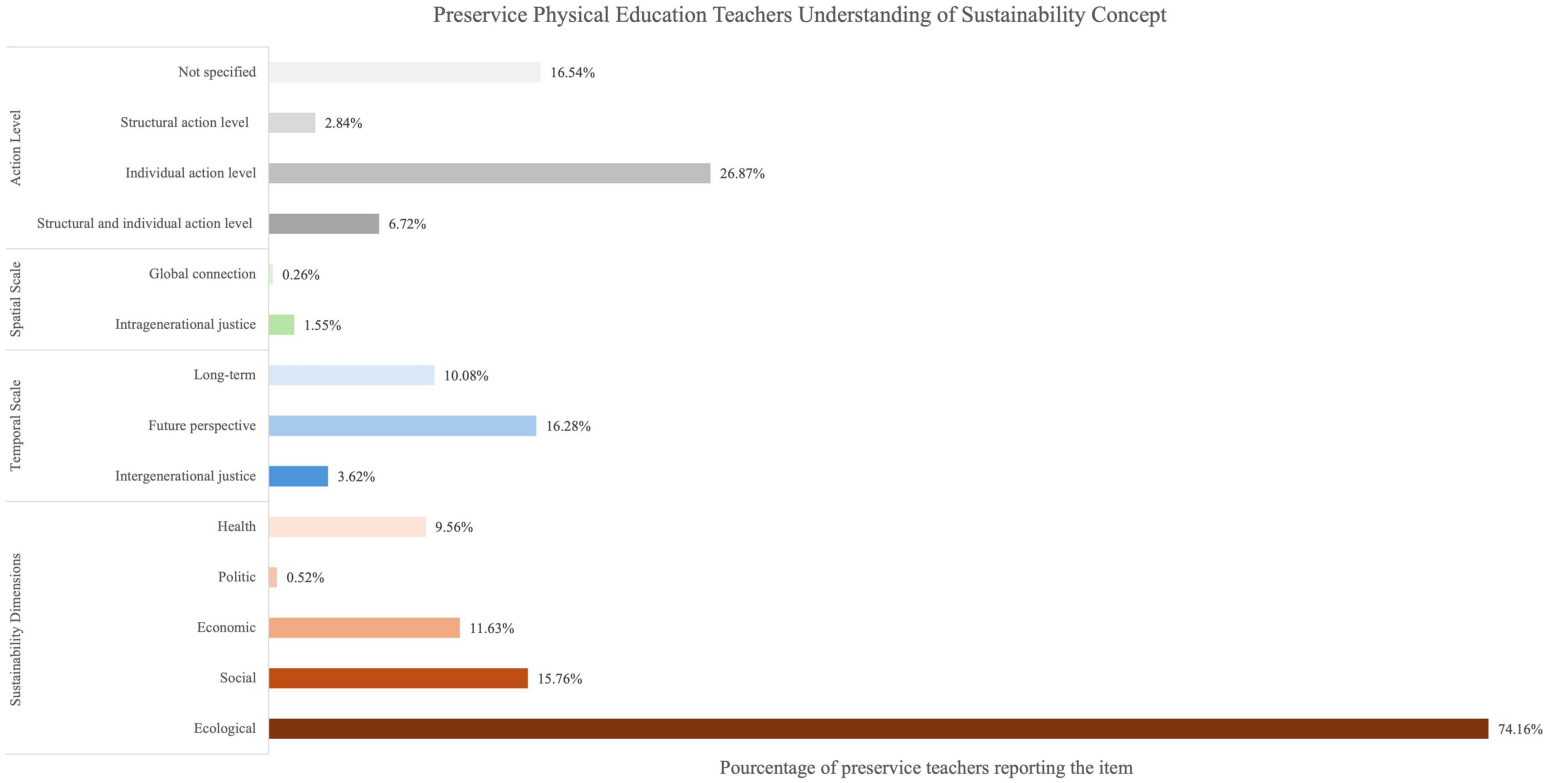
Preservice physical education teachers understanding of sustainability concept.

**Figure 5:**
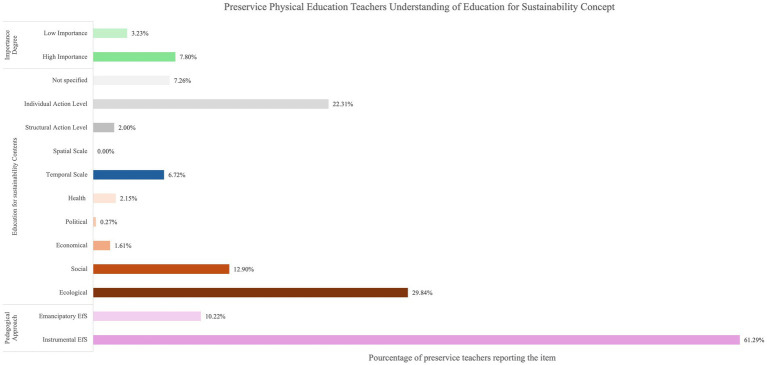
Preservice physical education teachers understanding of education for sustainability concept.

**Figure 6:**
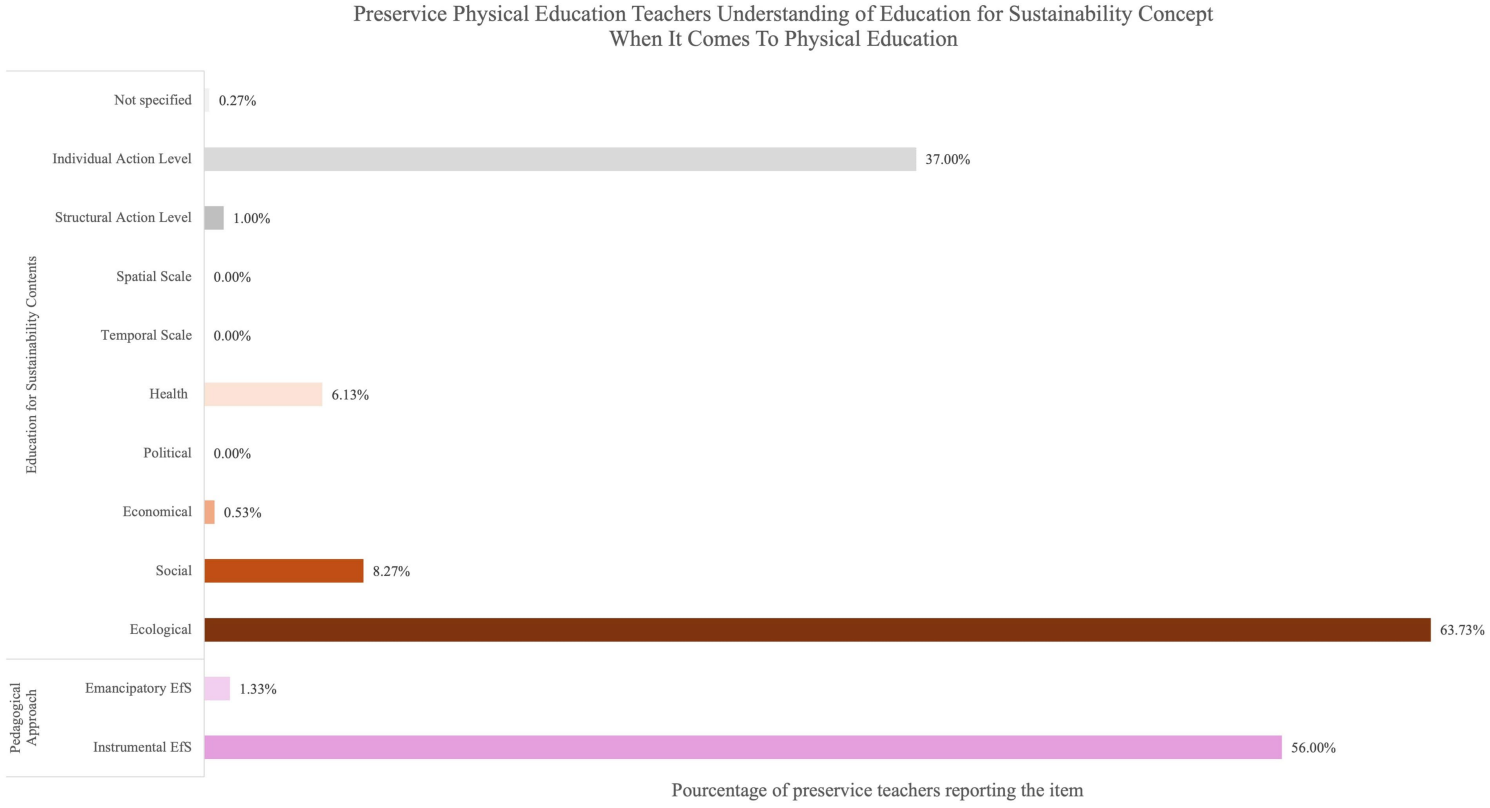
Preservice physical education teachers understanding of education for sustainability concept when it comes to physical education.

**Figure 7:**
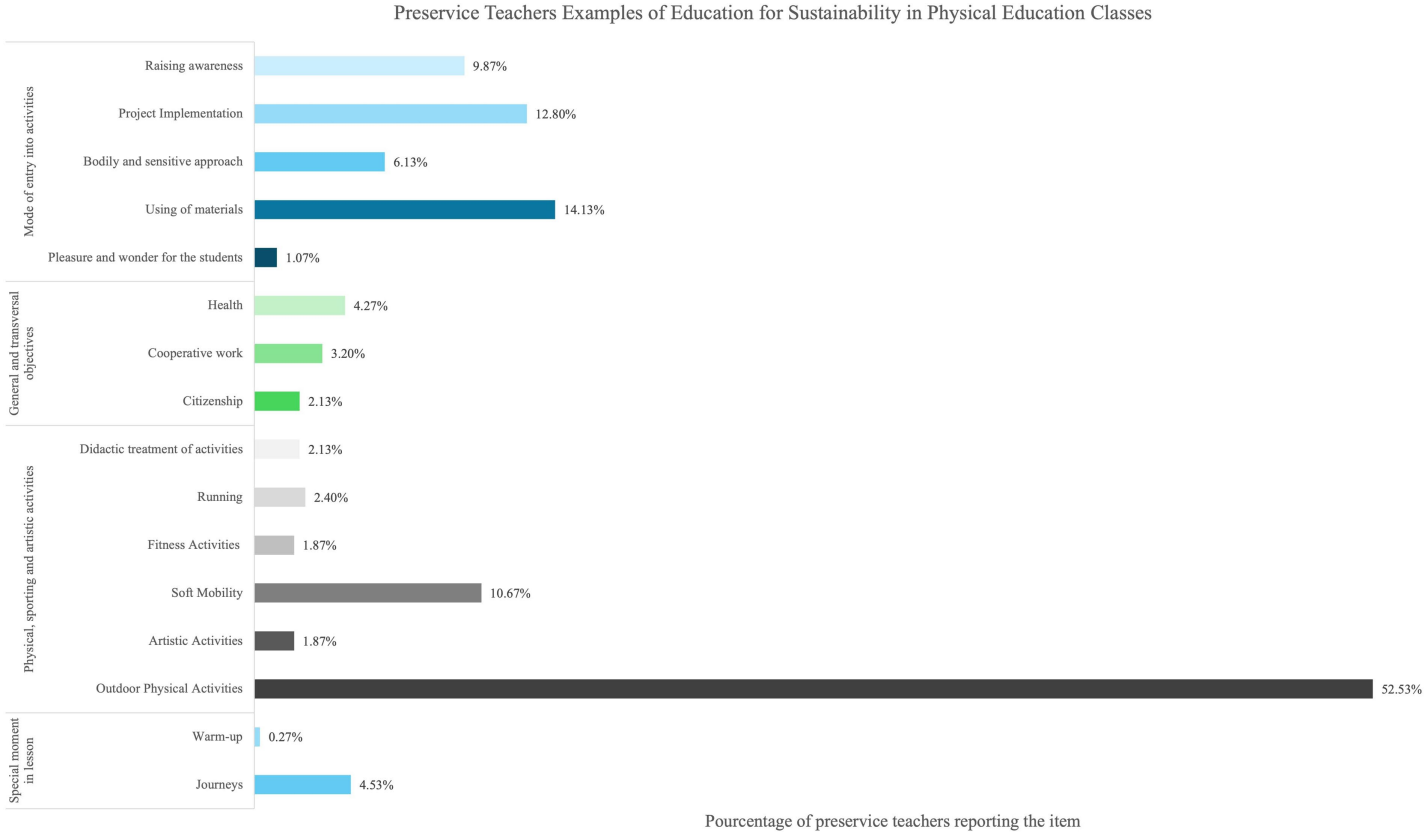
Preservice physical education teachers examples of education for sustainability in physical education classes.

The thematic analysis of the responses to the open-ended questions and the quantification of the items are presented in Figures 4–7. When preservice teachers were asked to associate words with the concept of sustainability (Figure 4), the ecological dimension was most frequently mentioned (74.16%), followed by individual action (26.87%). The temporal scale indicated that teachers associated sustainability with long-term (10.08%) and future perspectives (16.28%). Notably, the spatial scale was scarcely mentioned (1.81%). When asked to associate words to define the concept of EfS (Figure 5), an instrumental approach was highlighted (61.29%). The content focused mainly on the ecological (29.84%), social (12.90%), and individual levels of action (22.31%). Finally, when the preservice teachers were asked to provide concrete examples of EfS in PE (Figure 6), the dominant instrumental approach (56.00%) again emphasized content related to the ecological dimension (63.73%) and individual levels of action (37.00%) (e.g.*, “encourage pupils to collect and sort rubbish properly by discussing the consequences of pollution on ecosystems”*). The examples included specific activities, notably outdoor activities (52.53%) (e.g., “*Create an orienteering sequence focusing on sustainable development, with objectives for each marker*”), and, to a lesser extent, soft mobility (10.67%). Additionally, specific strategies for addressing EfS in PE were highlighted, such as reflecting on material use (14.13%) (e.g., “*Try to repair the chasubles and equipment in general before changing them*”), implementing projects (12.80%) (e.g., “*Organize an educational cycling trip in collaboration with the science teacher*”), and raising student awareness (9.87%) (e.g., “*Outdoor physical education courses can include activities such as hiking, camping, kayaking or cycling that allow students to develop a love and respect for nature while learning to minimize their environmental impact*”) (Figure 7).

### Preservice physical education teachers’ professional action competence in education for sustainability profiles

The results of the LPA are presented in Table 2. The AIC, BIC, ABIC, and LRT results indicated that the four class models exhibited the best fit. Specifically, decreases were observed between two, three and four classes with respect to the AIC, BIC, and ABIC results but not between four and five classes. LRT also revealed that four classes exhibited a better fit than did three classes, whereas five classes did not exhibit a better fit than did four classes. On the basis of the interpretability of the profiles and the statistical indicators in the LPA, a four-class solution was preferred for the PACeS profiles.

**Table 2:** Fit indices of latent profile analyses with profiles 1–5.

Number of classes	1	2	3	**4**	5
Number of parameters	6	10	14	**18**	22
Log likelihood	−1475.426	−1332.423	−1296.095	**−1265.067**	−1251.361
AIC	2962.851	2684.846	2620.191	**2566.133**	2546.722
BIC	2986.977	2725.056	2676.485	**2638.512**	2635.185
Sample size adjusted BIC	2967.938	2693.324	2632.060	**2581.394**	2565.374
LMRT	–	274.603*	72.655*	62.057*	27.411
Entropy	–	0.739	0.764	**0.797**	0.838

The bold entries reflect the selected model. *p < 0.05. AIC = Akaike information criterion; BIC = Bayesian information criterion; ABIC = adjusted BIC; LMRT = Lo, Mendell, and Rubin likelihood ratio test.

The PAC profiles are presented in Table 3. The descriptive labels for the four profiles thus identified were as follows: (a) a high profile (*n* = 29, including 10 females and 19 males), in which preservice PE teachers reported high scores on SEesd (*β* = 5.49, SD = 0.11), pPCKesd (*β* = 5.54, SD = 0.09), and Wesd (*β* = 4.35, SD = 0.21) in comparison with the remaining sample; (b) a moderate-high score profile (*n* = 188, including 76 females and 111 males), in which preservice PE teachers reported moderate-high scores on SEesd (*β* = 4.68, SD = 0.05), pPCKesd (*β* = 4.80, SD = 0.06), and Wesd (*β* = 3.44, SD = 0.07) in comparison with the remaining sample; (c) a low-moderate score profile (n = 164, including 49 females and 115 males), in which preservice PE teachers reported moderate-low scores on SEesd (*β* = 4.04, SD = 0.06) and pPCKesd (*β* = 3.87, SD = 0.05) as well as low scores on Wesd (*β* = 2.65, SD = 0.08) in comparison with the rest of the sample; and (d) a low profile (*n* = 31, including 6 females and 25 males), in which pre-service PE teachers reported low scores on SEesd (*β* = 3.07, SD = 0.14), pPCKesd (*β* = 3.03, SD = 0.16), and Wesd (*β* = 2.21, SD = 0.16) in comparison with the rest of the sample. A complementary MANOVA revealed that the scores on pPCKesd, SEesd, and Wesd differed significantly (*p* < 0.001) among the groups (high profile > moderate-high profile > low-moderate profile > low profile). Levene’s tests indicated no significant violations of the homogeneity of variances assumption across group comparisons (*p* > 0.05) supporting the use of MANOVA.

**Table 3:** Profiles of physical education preservice teachers.

Variables	High profile Mean (SD)	Moderate-high profile Mean (SD)	Low-moderate profile Mean (SD)	Low profile Mean (SD)
SEesd	5.59 (0.31)	4.70 (0.41)	4.03 (0.40)	3.01 (0.50)
pPCKesd	5.63 (0.26)	4.81 (0.39)	3.84 (0.35)	2.98 (0.59)
Wesd	4.27 (0.89)	3.47 (0.70)	2.63 (0.73)	2.15 (0.84)

SEesd = Self-efficacy regarding education for sustainability (EfS); pPCKesd = Perceived Pedagogical Content Knowledge regarding EfS; Wesd = Willingness for EfS.

The quantification of the thematic analyses of the responses to the open-ended questions by profile and the associated chi-square tests are detailed in Tables 4–6. With respect to the concept of sustainability, chi-square tests revealed significant differences between profiles (Table 4). Specifically, the high profile emphasized a greater level of individual and structural action than did the moderate–high (*χ*^2^ = 5.98; *p* = 0.014) and low-moderate profiles (*χ*^2^ = 13.3; *p* < 0.001). Additionally, the low-moderate profile mentioned the ecological dimension more frequently than did the moderate–high profile did (*χ*^2^ = 9.5; *p* = 0.002). No other significant difference in the definition of sustainability was identified between the profiles.

**Table 4:** Thematic analysis of sustainability definitions: chi-square test and response quantification.

		All profiles								Profile vs. Profile
		*χ* ^2^	ddl	*p*-value	“Yes”/"no”	*N* = 387				
						High profile	Moderate-high profile	Low-moderate profile	Low profile	
Dimensions	Ecological	10.7	3	0.013*	Yes	20	118	127	22	Moderate-Low > Moderate-High**
					No	9	58	28	5	
	Social	2.99	3	0.393	Yes	7	24	24	6	
					No	22	152	131	21	
	Economical	1.97	3	0.579	Yes	2	18	22	3	
					No	27	158	133	24	
	Political	2.41	3	0.492	Yes	0	2	0	0	
					No	29	174	155	27	
	Health	0.74	3	0.864	Yes	3	15	17	2	
					No	26	161	138	25	
Temporal scale	Intergenerational justice	1.44	3	0.697	Yes	2	7	4	1	
					No	27	169	151	26	
	Futures perspectives	3.67	3	0.299	Yes	8	29	21	5	
					No	21	147	134	22	
	Long term	5.32	3	0.150	Yes	2	12	22	3	
					No	27	164	133	24	
Spatial scale	Intragenerational justice	1.37	3	0.712	Yes	0	3	2	1	
					No	29	173	153	26	
	Global connection	1.50	3	0.682	Yes	0	0	1	0	
					No	29	176	154	27	
Action level	Individual and structural	12.9	3	0.005**	Yes	6	12	5	3	High > Moderate-High* High > Moderate-Low***
					No	23	164	150	24	
	Individual	1.60	3	0.660	Yes	5	50	42	7	High > Moderate-Low*
					No	24	126	113	20	
	Structural	7.16	3	0.067	Yes	3	5	3	0	
					No	26	171	152	27	
	Not specified	2.17	3	0.537	Yes	3	34	23	4	
					No	26	142	132	23	

**Table 5:** Thematic analysis of education for sustainability definitions: chi-square test and response quantification.

		All profiles								Profile vs. Profile
		*χ* ^2^	ddl	*p*-value	“Yes”/"no”	*N* = 372				
						High profile	Moderate-high profile	Low-moderate profile	Low profile	
Pedagogical approach	Instrumental EfS	5.45	3	0.142	Yes	15	101	91	21	
					No	14	68	57	5	
	Emancipatory EfS	1.8	3	0.615	Yes	3	21	12	2	
					No	26	148	136	24	
Contents	Ecological	11.8	3	0.008**	Yes	7	43	46	15	Low > High* Low > Moderate-High*** Low > Moderate-Low**
					No	22	126	102	11	
	Social	7.72	3	0.052	Yes	6	28	13	1	Moderate-High > Moderate-Low*
					No	23	141	135	25	
	Economical	16.8	3	<0.001***	Yes	3	3	0	0	High > Moderate-High * High > Moderate-Low***
					No	26	166	148	26	
	Political	1.2	3	0.752	Yes	0	1	0	0	
					No	29	168	148	26	
	Health	10.6	3	0.014*	Yes	3	2	3	0	High > Moderate-High** High > Moderate-Low*
					No	26	167	145	26	
	Temporal Scale	2.03	3	0.566	Yes	2	12	11	0	
					No	27	157	137	26	
	Spatial Scale	/	3	/	Yes	0	0	0	0	
					No	0	0	0	0	
	Individual Action Level	5.41	3	0.144	Yes	2	37	36	8	
					No	27	132	112	18	
	Structural Action Level	8.95	3	0.03*	Yes	3	3	2	1	High > Moderate-High** High > Moderate-Low*
					No	26	166	146	25	
	Not specified Action Level	10	3	0.019*	Yes	6	11	10	0	High > Moderate-High* High > Moderate-Low*
					No	23	158	138	26	

**Table 6:** Thematic analysis of education for sustainability implementations in physical education: chi-square test and response quantification.

			All profiles							
			*χ* ^2^	ddl	*p*-value	“Yes”/"no”	*N* = 375			
							High profile	Moderate-high profile	Low-moderate profile	Low profile
Pedagogical approach	Instrumental EfS		1.99	3	0.575	Yes	13	96	87	14
						No	16	76	61	12
	Emancipatory EfS		2.76	3	0.43	Yes	1	2	1	1
						No	28	170	147	25
General EfS contents	Ecological		1.29	3	0.732	Yes	16	113	93	17
						No	13	59	55	9
	Social		2.88	3	0.41	Yes	3	15	9	4
						No	26	157	139	22
	Economical		0.359	3	0.949	Yes	0	1	1	0
						No	29	171	147	26
	Political		/	3	/	Yes	0	0	0	0
						No	29	172	148	26
	Health		2.19	3	0.533	Yes	2	10	11	0
						No	27	162	137	26
	Temporal scale		/	3	/	Yes	0	0	0	0
						No	29	172	148	26
	Spatial scale		/	3	/	Yes	0	0	0	0
						No	29	172	148	26
	Individual action level		3.43	3	0.329	Yes	8	60	63	9
						No	21	112	85	17
	Structural action level		6.34	3	0.096	Yes	0	1	0	1
						No	29	171	148	25
	Not specified action level		1.18	3	0.757	Yes	0	1	0	0
						No	29	171	148	26
PE specific EFS contents	School trips		1.69	3	0.64	Yes	1	15	10	1
						No	28	157	138	25
	Sportive association		0.295	6	0.961	Yes	1	8	8	1
						No	28	164	140	25
	Special moment in lesson	Journeys	4.39	3	0.223	Yes	2	5	7	3
						No	27	167	141	23
		Warm-up	12	3	0.008**	Yes	1	0	0	0
						No	28	172	148	26
	Physical, sporting and artistic activities	Outdoor physical activities	0.418	3	0.937	Yes	14	90	80	13
						No	15	82	68	13
		Artistic activities	4.66	3	0.198	Yes	0	6	1	0
						No	29	166	147	26
		Soft mobility	1.22	3	0.748	Yes	4	16	18	2
						No	25	156	130	24
		Fitness activities	1.26	3	0,738	Yes	0	4	3	0
						No	29	168	145	26
		Running	2.34	3	0.505	Yes	1	6	2	0
						No	28	166	146	26
		Didactic treatment of activities	3.85	3	0.278	Yes	2	3	3	0
						No	27	169	145	26
	General and transversal objectives	Citizenship	8.21	3	0.042*	Yes	2	2	2	2
						No	27	170	146	24
		Cooperative work	2.02	3	0.569	Yes	2	6	3	1
						No	27	166	145	25
		Health	1.74	3	0.627	Yes	2	7	7	0
						No	27	165	141	26
	Mode of entry into activities	Pleasure and wonder for the students	4.77	3	0.189	Yes	0	4	0	0
						No	29	168	148	26
		Using of materials	1.47	3	0.69	Yes	5	27	17	4
						No	24	145	131	22
		Bodily and sensitive approach	0.657	3	0.883	Yes	2	10	11	1
						No	27	162	137	25
		Project implementation	2.23	3	0.526	Yes	6	23	16	3
						No	23	149	132	23
		Raising awareness	3.63	3	0.305	Yes	3	18	11	5
						No	26	154	137	21

With respect to the concept of EfS, chi-square tests revealed numerous significant differences between profiles (Table 5). The high profile placed greater emphasis on structural levels of action than did the moderate–high (*χ*^2^ = 8.95; *p* = 0.03) and low-moderate profiles (*χ*^2^ = 6.19; *p* = 0.013). The high profile also exhibited a higher unspecified level of action than did the moderate–high (*χ*^2^ = 6.34; *p* = 0.012), moderate–low (*χ*^2^ = 5.72; *p* = 0.017), and low profiles (*χ*^2^ = 6.04; *p* = 014). Furthermore, the high profile included more dimensions in EfS content, such as health and economic dimensions, than did the moderate–high (*χ*^2^ = 8.44; *p* = 0.004; *χ*^2^ = 6.19; *p* = 0.013) and low-moderate profiles (*χ*^2^ = 5.12; *p* = 0.024; *χ*^2^ = 15.6; *p* < 0.001). The moderate–high profile mentioned the social dimension more frequently than did the low-moderate profile did (*χ*^2^ = 4.45; *p* = 0.039). Finally, the low profile mentioned the ecological dimension more frequently than did the high (*χ*^2^ = 6.43; *p* = 0.011), moderate-high (*χ*^2^ = 11.2; *p* < 0.001), and low-moderate profiles (*χ*^2^ = 6.88; *p* = 0.009).

With respect to the implementation of EfS in PE, chi-square tests revealed only two differences between profiles: the use of the warm-up as a privileged moment for EfS and the use of citizenship work as a disciplinary entry point (Table 6). However, the marginal number of mentions of these elements led to the identification of these results as not significant. Otherwise, no differences in pedagogical approaches to EfS or in the general content of EfS were identified in the examples provided by the participants.

## Discussion

### Preservice physical education teachers’ professional action competence in the process of implementing education for sustainability

The first objective of this research was to evaluate the PACesd among preservice PE teachers. The results revealed a higher mean score for perceived PCKesd and a lower mean score for Wesd than was the case in the results reported by Sass et al. (2022) with teachers of several subjects in Belgium. The results also highlighted gaps between perceived PCKesd and the external assessment of the PCKesd.

First, preservice PE teachers obtained moderate–high scores in terms of perceived PCKesd (PCKesd = 4.35; SD=0.78). The mean pPCKesd scores obtained for the preservice PE teachers included in the sample were significantly higher than the scores reported by Sass et al. (2022) (pPCKesd = 4.26; SD = 0.78). The external evaluation of PCKesd, which is based on the responses to the open-ended questions, nuanced the self-reported results. Preservice PE teachers showed a limited understanding of sustainability and EfS concepts, focusing mainly on the ecological dimension while rarely emphasizing its holistic nature. Although the temporal scale was sometimes mentioned, the spatial scale and structural levels of action were largely overlooked, with most responses centered on individual responsibility. These findings highlight an incomplete grasp of sustainability’s complex, multidimensional character (Raworth, 2017).

Regarding EfS, preservice PE teachers mainly adopted an instrumental approach focused on promoting eco-friendly behaviors through outdoor activities, contrasting with the emancipatory approach emphasized in the literature (Sass et al., 2020; Wals et al., 2008). The ecological dimension and individual action remained dominant, as reflected in examples like picking up waste during orienteering. These findings suggest that PE teachers currently lack sufficient PCK to implement an emancipatory EfS model that fosters students’ sustainability action competences (Sinakou et al., 2019).

These results are consistent with studies investigating PCK in the context of PE. PE teachers exhibited inaccuracy and vagueness in concepts related to sustainability and EfS (Lohmann and Goller, 2023; Merma-Molina et al., 2023). In line with our study, PE teachers were not able to register the multidimensionality of sustainability, instead mainly highlighting the environmental perspective (Baena-Morales et al., 2022). The discrepancy between perceived PCK and external assessment is also not surprising. These findings indicate a gap between the level of PCK that preservice PE teachers believed they possessed and their actual level. These results are in line with those of previous studies that highlighted that preservice middle school teachers’ knowledge of EfS is not related to their personal teaching self-efficacy with respect to sustainability (Stants, 2016). A similar trend was reported in a recent paper on teacher trainers (Castéra et al., 2020). French teacher trainers exhibit significantly more confidence in their PCK than do their counterparts from elsewhere in Europe or Asia. The authors of that paper attributed this outcome to the long-established tradition of *didactique* in French teacher education. These results indicate a need for training on the concepts of sustainability and EfS for preservice PE teachers to implement EfS effectively in PE, both generally and within the specific context of PE.

Second, preservice PE teachers obtained moderate-high scores of SEesd with respect to their ability to implement EfS in the context of PE (SEesd = 4.36; SD = 0.71). The mean SEesd score for the preservice PE teachers included in this sample did not differ significantly from the results reported by Sass et al. (2022) (SEesd = 4.38; SD = 0.68), with a sample of teachers across several disciplines. This study was the first to investigate preservice PE teachers’ self-efficacy in the particular context of EfS. Nevertheless, studies have investigated the perceived competence of PE teachers in integrating sustainability-related content into their teaching (Baena-Morales et al., 2023a; Froberg et al., 2022; Wiklander et al., 2024). Baena-Morales et al. (2023a) highlighted the fact that preservice PE teachers in the Spanish context exhibit high-level self-perceptions of competencies related to sustainable development as well as the three dimensions of this factor (environmental, social, and economic). These results have been confirmed in the Swedish context by reference to certified upper secondary PE teachers (Wiklander et al., 2024) and certified PE teachers in preschool and compulsory school (Froberg et al., 2022), in which context high levels of self-perceived competence were also highlighted. Furthermore, it was shown that young adults, like our participants, were those whose representations of the concept of sustainability were closest to the definition of the concept (Barone et al., 2020). As the difference between sustainability and EfS is not easy for PE teachers, it is possible that this may have led them to indicate a moderate to high level of competence. Nevertheless, these findings concerning the moderate–high levels of self-efficacy exhibited by preservice PE teachers in the context of EfS are still somewhat surprising, as the explicit connections between EfS and PE are recent (Baena-Morales and Gonzalez-Villora, 2023). Previous research has highlighted a relative lack of sustainability or EfS in the PE curriculum (Froberg et al., 2023; Olive and Enright, 2021), and few PE teachers have reported teaching their pupils about sustainability in their classes (Froberg et al., 2022). Previous studies have also indicated that PE teachers perceive that they need professional development in the context of sustainability and EfS (Froberg et al., 2022; Lohmann and Goller, 2023). The moderate–high levels of self-efficacy observed among preservice PE teachers with respect to the implementation of EfS in PE classes could be explained by the Dunning–Krueger effect (Dunning, 2011; Kruger and Dunning, 1999). This framework explains overconfidence by referring to the fact that low-information individuals suffer from various gaps and errors that lead them to make many mistakes, of which they remain unaware. This point remains hypothetical, and direct observations of EfS teaching sequences in PE classes should supplement these self-reported data.

Finally, with respect to the first objective, preservice PE teachers obtained moderate–low scores in terms of their willingness to implement EfS in PE (Wesd = 3.09; SD = 0.92). The mean Wesd score for the preservice PE teachers included in the sample was significantly lower than the scores reported by Sass et al. (2022) (Wesd = 3.32; SD = 1.00). These results were in line with those of a recent study conducted by Lorente-Echeverría et al. (2024), but they partially disagreed with the results of other studies conducted in the German context, which highlighted PE teachers’ positive attitudes toward EfS (Lohmann and Goller, 2023; Lohmann et al., 2023). This lack of consensus has also been observed in studies that have investigated teachers in other disciplines. Most such studies have highlighted the fact that teachers exhibit positive attitudes toward the possibility of implementing sustainability and EfS in their teaching (Anyolo et al., 2018; Burmeister et al., 2013; Park et al., 2016; Pegalajar-Palomino et al., 2021). Nevertheless, previous research has highlighted that all teachers are not equally willing to implement EfS (Goller and Rieckmann, 2022; Malik et al., 2023). These results highlight the need to explore the barriers identified by preservice PE teachers regarding the implementation of EfS in PE classes.

### Preservice physical education teachers’ profiles about professional action competence in the implementation of education for sustainability

The second objective of this study was to establish profiles based on professional action competence levels in the process of implementing EfS among preservice PE teachers. The results highlighted four profiles of PE teachers based on their levels of PAC: (a) the high score profile; (b) the moderate–high score profile; (c) the low-moderate score profile; and (d) the low score profile. In line with a previous study that investigated teachers’ interest in EfS (Sinakou et al., 2024) this finding confirms the interindividual variability among PE teachers in terms of PACesd. Notably, most of the participants in this research were assigned to the moderate–high profile (*n* = 188) or the low-moderate profile (*n* = 164), whereas the two extreme profiles were least common in the present sample. Most preservice PE teachers do not seem to exhibit particularly positive or negative perceptions regarding EfS. The three scales that composed the PACesd questionnaire were also highly correlated. These results could explain why all these profiles were organized in a similar pattern, such that the level of willingness was lower than the levels of self-efficacy and PCK.

The thematic analysis by profile has clarified the different profiles in terms of PCK. Notably, the high profile demonstrated a better understanding of sustainability and EfS concepts.

The high profile of preservice PE teachers emphasized the need for both structural and individual action to address sustainability issues, which was reflected in their EfS content. Compared with other profiles, they also highlighted more dimensions, including health and economic aspects, in EfS learning content. Overall, the high and moderate-high profiles addressed more dimensions than the low to moderate and low profiles did, with the low profile focusing almost exclusively on the ecological dimension. These results are in line with those of the study by Lohmann and Goller (2023), which revealed that PE teachers endorse a wide range of subjective theories regarding sustainability and EfS. However, these differences require nuance. No differences were detected in the pedagogical approach. All the profiles emphasized an instrumental approach, teaching simple eco-friendly behaviors. All the profiles also struggled to grasp the temporal and spatial scales of sustainability, particularly with respect to EfS content. Finally, no significant differences were found between profiles in implementing EfS in PE. This result is notable from a training perspective, as it indicates that preservice PE teachers, regardless of their understanding of sustainability and EfS concepts, struggle to move beyond an instrumental model focused on transmitting eco-friendly behaviors in outdoor activities. In light of these findings, general training in sustainability and EfS concepts appears insufficient. It seems necessary to specifically address EfS within the context of PE in the training of PE teachers (Baena-Morales et al., 2023b; Lohmann and Goller, 2023).

## Limitations and perspectives

An initial limitation of this research was that the PACesd questionnaire results were not cross-referenced with other self-reported data. In particular, it would be interesting to cross-reference the results of the questionnaire with data concerning eco-anxiety (Ágoston et al., 2022), teachers’ levels of expertise (Lentillon-Kaestner et al., 2024), professional identity (Hanna et al., 2020), or value orientation (Drouet et al., 2021).

Second, as highlighted by Sass et al. (2022), the questionnaire relied solely on self-reported data. While triangulation with an external evaluation of PCK is an important step in consolidating the conclusions of this study, it should be complemented by field observations. This last step could enable a precise evaluation of the PACesd of preservice PE teachers.

Third, as with most studies that include self-reported data from questionnaire responses involving social norms, the results of this study might be affected by social desirability bias (van de Mortel, 2008).

Finally, for a few variables of the chi-square tests (e.g., economical and political dimensions, temporal and spatial scales), some expected cell frequencies fell below the conventional criteria, which may affect the robustness of the chi-square results. These cases were interpreted with appropriate caution and would benefit from further investigation through larger sample sizes or complementary qualitative approaches, such as semi-structured interviews.

The results of this study suggest several applied perspectives for both future research and the training of PE teachers. First, with respect to our fit indicators, the use of the French translated version of the PACesd questionnaire seems to be relevant for further studies in the context of PE. Second, the significant correlations among all the scales included in the PACesd questionnaire could provide valuable support for both the initial training and the in-service training of PE teachers. These results suggest that different approaches could improve the overall PAC of PE teachers. For example, addressing factors that hinder the desire to implement (willingness) or the construction of ways of teaching PCK that are linked to the implementation of EfS in PE could be beneficial. Improving one of these scales may positively impact the other two scales of PACesd. Explorations of these options could facilitate the differentiation and individualization of training approaches based on the teacher profiles encountered in training courses. Third, the relatively low level of willingness to implement EfS in PE observed among the sample investigated in this research highlights the need to explore the major barriers identified by future PE teachers. Addressing these barriers is crucial for the implementation of effective EfS with students as well as with respect to designing training courses that can support PE teachers more effectively. Finally, as noted by Sass et al. (2022) regarding the use of the PACesd questionnaire, this exploratory study could serve as a pretest that can facilitate the subsequent development of the PACesd among future PE teachers who must still be identified. This approach could be valuable for evaluating the effectiveness of a training process in the context of EfS in PE or from the perspective of professional training.

## Conclusion

This study explored preservice PE teachers’ professional action competence in implementing education for sustainability (EfS). The results revealed moderate-high self-efficacy and perceived pedagogical content knowledge (PCK) but a lower willingness to implement EfS. The external PCK assessment revealed a partial understanding of sustainability and EfS concepts, highlighting discrepancies between self-reported and observed data. This emphasizes the value of mixed methods research for capturing complexity beyond quantitative self-reports. Four competence profiles emerged, showing variability in readiness to implement EfS, with most teachers in moderate-to-high or low-to-moderate categories. The high and moderate-high profiles demonstrated better conceptual understanding, but no differences were observed in EfS pedagogical approaches or implementation in PE. Despite these differences, common elements across profiles suggest considerations for teacher training. This study underscores the current state of EfS in PE and the need for further research on barriers to integration. More objective competence measures and classroom observations are recommended to bridge the gap between perceived and actual EfS teaching abilities.

## Data Availability

The raw data supporting the conclusions of this article will be made available by the authors, without undue reservation.
