# Supplementary material for: Preservice physical education teachers’ professional action competence in education for sustainability: a mixed method research
Source: Front Psychol. 2025 Jun 25;16:1601026. doi: 10.3389/fpsyg.2025.1601026 (PMC12239833; doi:10.3389/fpsyg.2025.1601026)
Supplement: Supplementary file 1 [file Table_1.docx]

**Appendice 1**

*French Version of the Professional Action Competence in Education for Sustainable Development Questionnaire*

| **Autoefficacité concernant l'éducation au développement durable**  *Je suis convaincu qu'en tant qu'enseignant, je peux…* | |
| --- | --- |
| SEesd1 | Développer la capacité des élèves à envisager un problème sous différents points de vue |
| SEesd2 | Développer la capacité des élèves à évaluer différentes solutions aux problèmes de durabilité |
| SEesd3 | Développer la capacité des élèves à réfléchir sur leurs propres actions. |
| SEesd4 | Développer la capacité des élèves à exprimer leur propre opinion sur les questions de durabilité |
| SEesd5 | Développer l'aptitude des élèves à comprendre l'interconnexion entre les aspects sociaux, environnementaux et économiques du développement durable |
| SEesd6 | Faire prendre conscience aux élèves qu'il existe des intérêts contradictoires en matière de développement durable. |
| SEesd7 | Faire prendre conscience aux élèves que les choix en matière de développement durable comportent un degré élevé d'incertitude |
| SEesd8 | Développer la capacité des élèves à agir en faveur du développement durable au niveau local (par exemple, dans l'école/établissement). |
| SEesd9 | Développer la capacité des élèves à agir pour le développement durable au niveau régional (par exemple, dans la municipalité). |
| SEesd10 | Développer la capacité des élèves à agir au niveau mondial en faveur du développement durable (par ex, boycotter certains produits). |
| **Perception de la connaissance du contenu pédagogique de l'éducation au développement durable**  *Je suis convaincu qu'en tant qu'enseignant, je peux…* | |
| pPCKesd1 | Faire en sorte que l'éducation au développement durable soit mise en œuvre dans ma (mes) classe(s). |
| pPCKesd2 | Faire de l'éducation au développement durable une réalité dans mon école/établissement. |
| pPCKesd3 | Évaluer un projet d'éducation au développement durable que j'ai (nous avons) mis en œuvre. |
| pPCKesd4 | Aborder les aspects environnementaux des problématiques de durabilité dans mon enseignement. |
| pPCKesd5 | Aborder les aspects sociaux des problématiques de durabilité dans mon enseignement. |
| pPCKesd6 | Aborder les aspects socioéconomiques des problématiques de durabilité dans mon enseignement. |
| pPCKesd7 | Aborder les aspects globaux des problématiques de durabilité dans mon enseignement |
| pPCKesd8 | Travailler sur le développement durable dans l'esprit d’objectifs à atteindre |
| pPCKesd9 | Travailler dans toutes les disciplines sur le développement durable |
| pPCKesd10 | Formuler des objectifs d'apprentissage pour mes étudiants en matière de développement durable |
| pPCKesd11 | Avoir la flexibilité de concevoir des environnements d'apprentissage pour travailler sur les questions de durabilité |
| **Volonté de mettre en œuvre l'éducation au développement durable**  *Je suis convaincu qu'en tant qu'enseignant, je peux…* | |
| Wesd1 | Chaque jour, je m'assure d'avoir suffisamment d'opportunités pour me consacrer à l'éducation au développement durable. |
| Wesd2 | L’éducation au développement durable, c'est typiquement moi. |
| Wesd3 | L’éducation au développement durable me tient à cœur. Sans l'éducation au développement durable, je ne serais pas moi-même. |
| Wesd4 | Mettre en œuvre l'éducation au développement durable me donne de l'énergie. |
| Wesd5 | J’essaie de planifier mon travail quotidien de manière à avoir le plus de temps possible à consacrer à l'éducation au développement durable. |
| Wesd6 | Quand je travaille sur l'éducation au développement durable, je le vis comme une expérience intense. |
| Wesd7 | L’éducation au développement durable jouera un rôle important dans ma vie. |
| Wesd8 | Je ressens souvent une forte envie de travailler l'éducation au développement durable |
| Wesd9 | Je suis souvent très impatient de travailler l'éducation au développement durable. |
| Wesd10 | Nombre de mes objectifs personnels sont liés à l'éducation au développement durable |
